# Evaluative Assay of Nuclear and Mitochondrial Genes to Diagnose *Leishmania* Species in Clinical Specimens

**Published:** 2017-10

**Authors:** Ahmad Reza ESMAEILI RASTAGHI, Adel SPOTIN, Mohammad Reza KHATAMINEZHAD, Mostafa JAFARPOUR, Elnaz ALAEENOVIN, Narmin NAJAFZADEH, Neda SAMEI, Neda TALESHI, Somayeh MOHAMMADI, Parviz PARVIZI

**Affiliations:** 1. Molecular Systematics Laboratory, Parasitology Department, Pasteur Institute of Iran, Tehran, Iran; 2. Dept. of Medical Parasitology & Mycology, Faculty of Medicine, Tabriz University of Medical Sciences, Tabriz, Iran; 3. Dept. of Biology, Tonekabon Branch, Islamic Azad University, Tonekabon, Iran

**Keywords:** *Leishmania* species, ITS-rDNA, Hsp70, Cyt *b*, Diagnosis, Iran

## Abstract

**Background:**

Leishmaniasis as an emerging and reemerging disease is increasing worldwide with high prevalence and new incidence in recent years. For epidemiological investigation and accurate identification of *Leishmania* species, three nuclear and mitochondrial genes (ITS-rDNA, Hsp70, and Cyt *b*) were employed and analyzed from clinical samples in three important Zoonotic Cutaneous Leishmaniasis (ZCL) foci of Iran.

**Methods:**

In this cross-sectional/descriptive study conducted in 2014–15, serous smears of lesions were directly prepared from suspected patients of ZCL in Turkmen in northeast, Abarkouh in center and Shush district in southwest of Iran. They were directly prepared from suspected patients and DNA was extracted. Two nuclear genes of ITS-rDNA, Hsp70 and one mitochondrial gene of Cyt *b* within *Leishmania* parasites were amplified. RFLP was performed on PCR-positive samples. PCR products were sequenced, aligned and edited with sequencher 4.1.4 and phylogenic analyses performed using MEGA 5.05 software.

**Results:**

Overall, 203 out of 360 clinical samples from suspected patients were *Leishmania* positive using routine laboratory methods and 231 samples were positive by molecular techniques. *L. major L. tropica*, and *L. turanica* were firmly identified by employing different molecular genes and phylogenic analyses.

**Conclusion:**

By combining different molecular genes, *Leishmania* parasites were identified accurately. The sensitivity and specificity three genes were evaluated and had more advantages to compare routine laboratory methods. ITS-rDNA gene is more appropriate for firm identification of *Leishmania* species.

## Introduction

Leishmaniasis is one of the most important human protozoan parasitic diseases worldwide by increasing the prevalence and incidence rates in recent years (1–3). From three types of leishmaniasis in Iran, Zoonotic Cutaneous Leishmaniasis (ZCL) has a great distribution reported from more than half of Iranian provinces (4, 5). ZCL is a single cell parasitic disease that considered as a major health problem in many areas of Iran.

Rodents and other mammals are reservoir hosts and human infects the causative agent of *Leishmania major* accidentally by biting female sand flies (6, 7). The presence of ZCL in Turkmen Sahara (Golestan), Abarkouh (Yazd), and Shush (Khuzestan) has been demonstrated in addition new ZCL foci in some locations located in border of Iran and Iraq (7, 8). *L. major, L. turanica, L. jerbilli* and a new *Leishmania* close to *L. Jerbilli* have been reported in ZCL foci but *L. major* is the principal agent of ZCL in Iran (3, 9, 10). *Phlebotomus papatasi* is the main vector and the most important reservoir hosts are *Rhombomys opimus* and *Meriones libycus* in Turkmen Sahara and Abarkouh, and *Tateraindica* in Shush (8, 11–13).

ZCL represents a typical model of emerging and reemerging zoonosis disease (14). ZCL can cause substantial morbidity because of the presence of a chronic skin ulcer and the psychological effect of disfigurement (15). There are no proper vaccines to protect people against the parasites. Although, epidemiological investigation and finding *Leishmania* species are helpful in the diagnosis of ZCL; however they are not sufficient methods to identify firmly the agent of the disease. Accurate identification of the causative agent of *Leishmania* species is essential to give us knowledge of the *Leishmania* species in the endemic and specific geographical area, and better approach in control measurements and treatment of disease (16). Hence, firmly identification and determination of *Leishmania* parasites are indispensable advanced molecular methods for human cases because of being deficient information for routine laboratory techniques.

For this investigation, three nuclear and mitochondrial genes (ITS-rDNA, Hsp70, and Cyt *b*) of *Leishmania* were employed, compared and designed to identify the *Leishmania* species parasites circulating in suspected patients of ZCL in different regions in Northeast, Central and Southwest to Iran.

## Materials and Methods

In this cross-sectional/descriptive study conducted in 2014–2015, serous smears of lesions were directly prepared from suspected patients of ZCL in Turkmen Sahara (37° 13′ 0″ N55° 0′ 0″ E) in northeast, Abarkouh (31° 7′ 44.04″ N 53° 16′ 56.64″ E) in center and Shush district (32° 11′ 39.12″ N 48° 14′ 36.96″ E) in southwest of Iran.

**Fig. 1:**
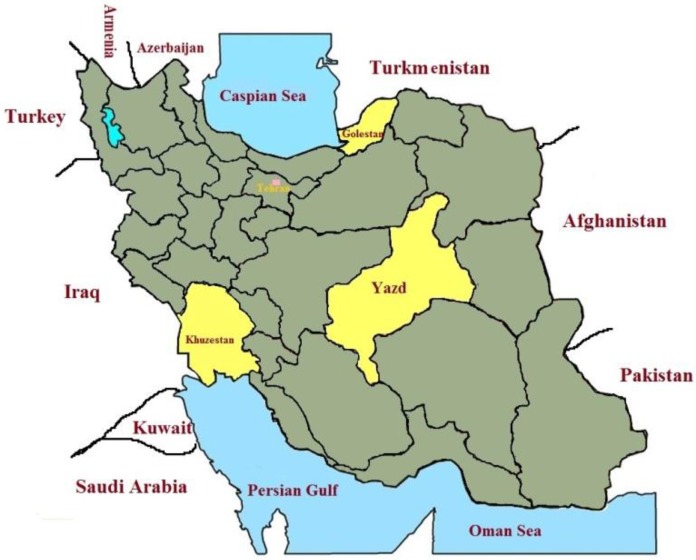
Iran map showing where sampling from suspected patients of ZCL took place

Human samples were collected from urban and rural areas surrounding Shush, Turkmen Sahara and Abarkouh districts of Iranian provinces. The personal information, lesion duration, type and number of lesion, ulcer(s)’ location, patients’ traveling to endemic regions and the grading numbers of amastigotes were recorded individually and kept confidential (Fig. 1).

Giemsa Stained slides were prepared. Amastigote presence in slides was observed and graded under light microscope (Table 1). DNA was extracted from the slides using Phenol-Chlorophorme method with minor modification (3).

**Table 1:** *Leishmania* identifications from suspected patients of three endemic ZCL foci, Iran

***Sampling site***			***Positive sample No.***	***Suspected patients NO***	***Microscopic Observation***	***Sex***		***Grading positive slides***						***Lesion form***		***Lesion No.***			***Lesion Site***			
**Provinces**	**Districts**	**Age Group**				**Male**	**Female**	**+1 1–10 Parasite / 1000 fields**	**+2 1–10 Parasite / 100 fields**	**+3 1–10 Parasite / 10 fields**	**+4 1–10 Parasite / field**	**+5 10–100 Parasite / field**	**+6 >100 Parasite / field**	**Wet**	**Dry**	**Single**	**Double**	**Many**	**Hand**	**Foot**	**Face & Noose**	**Other**
**Yazd**	**Abarkouh**	1–5	5	90	44	31	20	5	6	9	14	13	3	41	10	30	15	16	26	12	7	6
		5–10	15																			
		10–25	20																			
		<25	10																			
**Golestan**	**Turkmen Sahara**	1–5	10	150	80	61	29	6	10	12	28	24	10	73	17	32	34	24	36	29	16	9
		5–10	25																			
		10–25	30																			
		<25	25																			
**Khuzestan**	**Shush**	1–5	8	120	79	46	44	5	8	14	30	25	8	69	21	35	25	20	31	39	10	10
		5–10	18																			
		10–25	40																			
		<25	24																			
	Total		231/360 (64.1%)		203	231		16	24	35	72	62	21	183	48	97	74	60	93	80	33	25
								7%	10%	15%	32%	27%	9%	79%	21%	42%	32%	26%	40%	35%	14%	11%
								231						231		231			231			

Two nuclear genes of ITS-rDNA (480 bp), Hsp70 (750 bp) and one mitochondrial gene of Cyt *b* (880 bp) within *Leishmania* parasites were amplified to detect any *Leishmania* infection among samples from suspected patients following the primers and protocols (16).

**Fig. 2:**
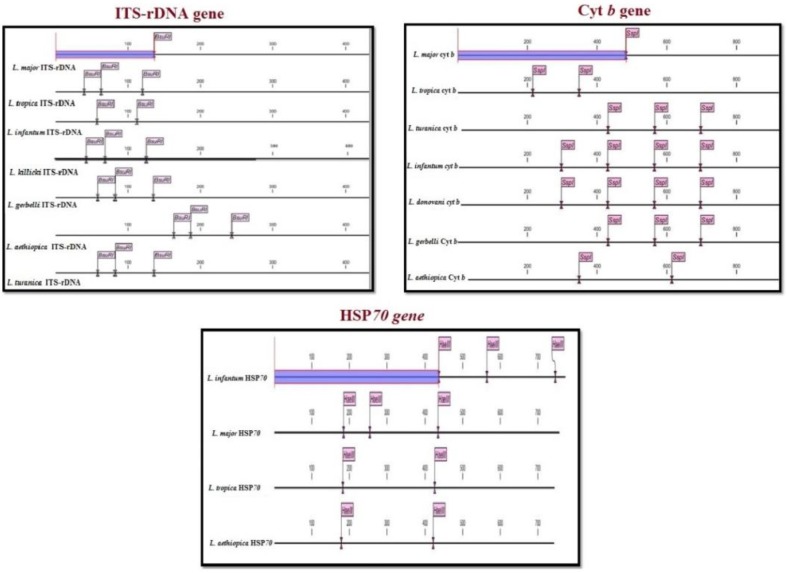
CLC DNA workbench software showing different digestion sites of BusrI and SspI enzymes effecting ITS-rDNA, Cyt b, and HSP70 genes In Sillico

To select suitable restriction enzyme, CLC DNA workbench 5.2 software (CLC bio A/S, Aarhus, Denmark) In Silico was employed. By choosing sequences of different *Leishmania* species registered in GenBank; enzyme *B*suRI (HaeIII) for ITS-rDNA and *S*spI enzyme for Cyt *b* gene were selected (Fig. 2). Hsp70 gene was not used for RFLP. Selected enzyme had different cut sites at GG↓CC in various species of *Leishmania* as a proper enzyme for PCR product digestion. RFLP was performed on PCR-positive samples for identification of *Leishmania* species. A master mix containing enzyme, Buffer and PCR product was prepared and stored at 37 °C for 4 hours.

**Fig. 3:**
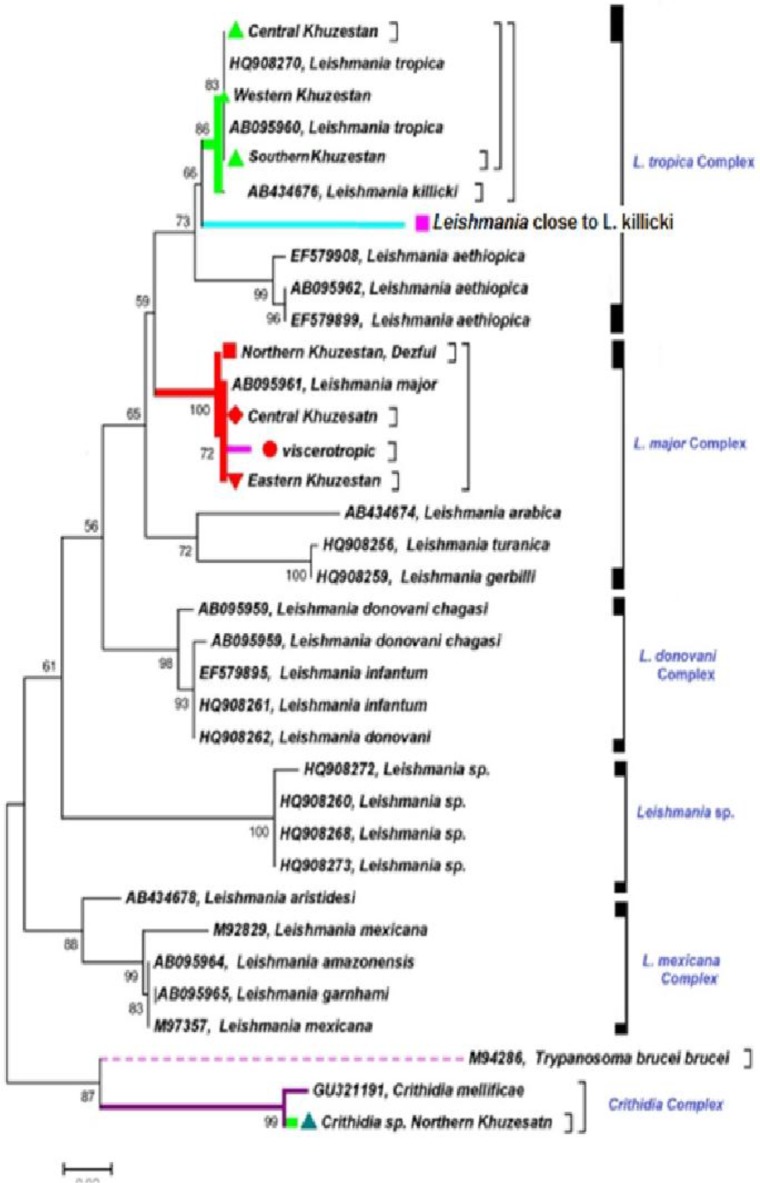
Maximum Likelihood algorithm showing the haplotypes of Cyt b gene for the isolates of Leishmania species using MEGA.5.05

Moreover, for accurate identification and confirmation of the specific PCR products were sequenced, aligned and edited with sequencher 4.1.4 software and phylogenic analyses was done using MEGA 5.05 software (Fig. 3).

## Results

The serous smears of lesions from 360-suspected patients (clinical samples) were sampled. Using routine laboratory methods, 203 samples from suspected patients were identified positive (Table 1). By employing molecular tools, using three different genes, 231 samples were *Leishmania* positive (Table 2, Fig. 2). After RFLP and/or sequencing; *L. major L. tropica* and *L. turanica* were firmly identified (Table 2).

**Table 2:** Different *Leishmania* species infection detected from suspected patients of ZCL from three endemic foci of Iran based on molecular markers

***Collection site***			***Microscopic Observation***	***Sex***		***Positive samples using molecular tools***	**Leishmania *positive via amplified genes***			***Molecular Methods***			
										**RFLP with *B*suRI& sequencing**			
**Provinces**	**Districts**	**Villages**		**Male**	**Female**		**ITS-rDNA**	**Hsp 70**	**Cyt b**	***L. major***	***L. tropica***	***L. turanica***	**Non-identified**
Yazd	Abarkouh	Abarghasr	44	20	31	51/90	30	10	11	48	4	0	2
		Chahgir											
		Harooni											
		Abarkouh											
Golestan	Turkmen	Kooran	80	29	61	90/150	45	20	20	85	0	2	3
	Sahara	Dashboroun											
		Gharegol											
		Hootan											
Khuzestan	Shush	Haft Tape	79	44	46	90/120	35	25	25	87	0	0	3
		Sorkhe											
		Aljazayer											
		Banader											
	Total		203/360	93	138	231/360	110/231 (47.6)	55/231 (23.8)	66/231 (28.5)	217/231 (93.9%)	4/231 (1.73%)	2/231 (0.86%)	8/231 (3.46%)
				231/360			231/360						

Shush district in south of Iran had higher infections than two other location where were sampled (87/360). The highest infection was in age group 10–25 yr (40/360) and male had more positive than females (Table 1).

Seventy-nine percent of the lesions were wet, 40% of them were in the hands, 42% of patients had single lesions and 32% of the slides were 4+, which was the highest velocity among the surveyed categories (Table 1).

Overall, 231 (64.1%) of samples were confirmed to be *Leishmania* positive via molecular methods (Table 2), of which 46.7%, 23.8%, and 28.5% were tested positive with by ITS-rDNA, Hsp70 and Cyt *b* genes, respectively (Table 2).

After RFLP and/or sequencing; from 231 *Leishmania* positive samples 217 (93.9%), 4 (1.73%) and 2 (0.86%) were indefinitely identified as *L. major L. tropica*, and *L. turanica,* respectively. In addition, 8 (3.46%) of the samples were unable to be accurately confirmed due to lack of PCR product and/or bad sequence reads (Table 2).

In this investigation, *L. major, L. tropica* and *L. turanica* were unambiguously typed and identified after analyzing and sequencing with molecular software with comparison to those sequences which have already been registered in GenBank in case of any similarity and homology. The obtained sequences of this investigation were homolog with *Leishmania* species after direct sequencing, editing, aligning and comparing with the sequences submitted to GenBank using Sequencher^TM^ 4.1.4.

In the present work, we have compared the specificity and sensitivity of three nuclear and mitochondrial genes, we discovered that sensitivity of ITS-rDNA was more than Cyt *b* and Hsp70 and ITS-rDNA show the highest specificity for *Leishmania* species.

## Discussion

In this investigation, *L. major, L. tropica* and *L. turanica* among suspected patients were firmly identified in three endemic ZCL foci, Iran. Three well-known molecular markers ITS-rDNA, Hsp70 and Cyt *b* genes were employed (3). Three genes were applied concurrently to compare specificity, sensitivity and to increase the chance of detecting any *Leishmania* parasites even in low concentrations (17).

ITS-rDNA gene is a nuclear, liner, homogenous and conserves gene with low intracellular polymorphism and readable sequences. ITS-rDNA is ideal for phylogenic analysis (12).

Cyt *b* as a mitochondrial marker can identify the novel nucleotide variations (haplotype) superior to ITS-rDNA (nucleus gene), this is associated with highly being conserved and high copy numbers of Cyt *b* per cell (20–50 maxi circles in 30 Kbp) (Fig. 2) (5, 18, 19).

ITS-rDNA shows high sensitivity because of approximately 20–400 copies of gene for *Leishmania* but not for differentiate *Leishmania* species. Cyt b as an evolutionary mitogenome marker has its semi conserved structure and low copy number could able to utilize in the discrimination of new mutants, whereas no significant mutant was observed in ITS-rDNA sequences (7).

By analyzing of CLC DNA workbench software, digestion sites of different enzymes were recognized on ITS-rDNA, Hsp70 and Cyt b genes of *Leishmania* species (Fig. 2). *B*usRI enzyme has one digestion site in *L. major* for ITS-rDNA gene that gives two fragments (120bp and 300 bp), three digestion sites for Hsp70 gene. *S*spI enzyme has one digestion site (∼ 500bp) for Cyt *b* gene in *L. major*. The different and variation fragments by digesting site make RFLP method, which generated species-specific patterns of bands visualized in agarose gels, a useful technique for accurate determination of *Leishmania* species.

Only amplifying DNA by PCR and observation of relevant band in agarose gels without sequences, molecular and phylogenetic analysis could not be effective and trusted firmly identification of *Leishmania* species (20).

Regarding data of this investigation, males are at higher risk than females for *Leishmania* infection in Iran because of wearing Hejab, covering skin by females, preventing sand fly bites and decreasing the risk for leishmaniasis. Moreover, men usually work in farms, fields and mostly sleep outside during the adult sand flies activity; these provide a good source of blood meals and transferring *Leishmania* parasites in ZCL foci (21). These also could be reason for high rate of infection in 10–25 age groups.

## Conclusion

Different genes combined for accurate identifications of clinical samples of *Leishmania* parasites sampled in three well-known ZCL foci in Iran. To find standardized, sensitive, specific, practical and reproducible genes for molecular identification and typing *Leishmania* species were evaluated and compared using three genes in this investigation. Molecular tools are more trustable than routine laboratory methods, and ITS-rDNA gene is more appropriate for accurate identification of *Leishmania* species.

## Ethical considerations

Ethical issues (Including plagiarism, informed consent, misconduct, data fabrication and/or falsification, double publication and/or submission, redundancy, etc.) have been completely observed by the authors.
